# INNOVATIONS IN LONG-TERM CARE SECTOR DURING COVID-19: A SCOPING REVIEW

**DOI:** 10.1093/geroni/igae098.2160

**Published:** 2024-12-31

**Authors:** Ruth Melo, Suzanne Santos, Sumaya Bhatti, Yanjun Duan, Charlene Chu, Franziska Zuniga, Lisa Cranley, Michael Lepore

**Affiliations:** University of São Paulo, São Paulo, Sao Paulo, Brazil; University of São Paulo, São Paulo, Sao Paulo, Brazil; University of Toronto, Toronto, Ontario, Canada; University of Toronto, Toronto, Ontario, Canada; University of Toronto, Toronto, Ontario, Canada; University of Basel, Basel, Basel-Stadt, Switzerland; University of Toronto, Toronto, Ontario, Canada; University of Massachusetts Amherst, Amherst, Massachusetts, United States

## Abstract

Different innovations were implemented in Long-Term Care (LTC) homes during COVID-19. Although designed to support stakeholders, innovations also have the potential to exacerbate disparities and inequalities in this sector. This scoping review aims to analyze innovations implemented in the residential LTC sector during the pandemic in four countries: Canada, USA, Brazil, and Switzerland. Potential studies were searched in six databases. Search strategy and eligibility criteria followed the mnemonic “PCC” (i.e., Population: residents, family members/caregivers, and other stakeholders, Concept: innovation, and Context: residential LTC sector). The studies retrieved from databases were screened by two independent reviewers. Discordances between reviewers were solved by consensus or by a third reviewer. A customized spreadsheet was used for data extraction. The search identified 4,056 registers. After excluding duplicate studies, 3,122 records were screened. From them, 98 studies fulfilled the eligibility criteria and were included. Half of the studies were conducted in the USA (51.0%), followed by Canada (39.8%). Few studies were conducted in Switzerland (n=3) and Brazil (n=1). Included studies have different design

**methods:**

qualitative (18.4%), observational (17.3%), experimental (17.3%) and mixed methods (10.2%). Other types of design methods (opinion, commentary, experience reports, editorial, etc.) and literature reviews accounted for 28.6% and 8.2%, respectively. The innovations presented in the studies were classified according to type, level, and setting. Stakeholder engagement with innovation was categorized as active, passive, and co-designed. results of studies that involved stakeholders are presented in the following papers.

